# Characterization of β‑lactoglobulin and κ‑casein genotypes PCR-RFLP in dairy cattle from Panama

**DOI:** 10.29374/2527-2179.bjvm001026

**Published:** 2026-07-24

**Authors:** Anabel Argelis García, Jenyfer Pérez García, Dilcia Sambrano, Fermín Acosta, César Edwards, José Torres, Diomedes Trejos, Amador Goodridge, Cecilia Escobar

**Affiliations:** 1 Facultad de Medicina Veterinaria, Universidad de Panamá, Panama City, Panama.; 2 Instituto de Investigaciones Científicas y Servicios de Alta Tecnología (INDICASAT AIP), Ciudad del Saber, Panama City, Panama.; 3 Sistema Nacional de Investigación (SNI), Secretaría Nacional de Ciencia, Tecnología e Innovación (SENACYT), Ciudad del Saber, Panama City, Panama.; 4 Instituto de Medicina Legal y Ciencias Forenses (IMELCF), Ciudad del Saber, Panama City, Panama.; 5 Ministerio de Desarrollo Agropecuario (MIDA), Panama City, Panama.

**Keywords:** κ-casein, β-lactoglobulin, PCR-RFLP, dairy cattle genetics, molecular markers, κ-caseína, β-lactoglobulina, PCR-RFLP, genética de bovinos leiteiros, marcadores moleculares

## Abstract

Sustained profitability in dairy production requires continuous improvements in milk yield and composition, traits that are strongly influenced by underlying genetic factors. Polymorphisms in the milk protein genes β-lactoglobulin (β-Lg) and κ-casein (κ-Cn) have been associated with variation in milk production, composition, and technological properties across dairy breeds. This study applied polymerase chain reaction-restriction fragment length polymorphism (PCR-RFLP) analysis to characterize β‑Lg and κ‑Cn gene variants in Jersey (JE), crossbred (XX), and Creole (CR) dairy cattle in Panama. Genotypic and allelic variation was evaluated using a convenience sample of 351 blood samples collected between 2014 and 2015 (JE, n = 230; XX, n = 90; CR, n = 26). Although the sampling strategy does not support population‑level inference, it is suitable for assessing the feasibility of PCR‑RFLP and conducting an exploratory characterization of genetic variability. Amplification success was 98.6% for β‑Lg and 76.6% for κ‑Cn. At the β‑Lg locus, allele A predominated in JE (0.55) and CR (0.52) cattle, whereas allele B was more frequent in XX cattle (0.73). At the κ‑Cn locus, allele B predominated across all groups. Overall, PCR‑RFLP proved to be a practical and accessible method for detecting variations in milk protein genes, supporting its application in future population‑based studies under Panamanian production conditions.

## Resumo

A rentabilidade sustentável da produção leiteira requer melhorias contínuas na produção e na composição do leite, características fortemente influenciadas por fatores genéticos subjacentes. Polimorfismos nos genes das proteínas do leite β-lactoglobulina (β-Lg) e κ-caseína (κ-Cn) têm sido associados a variações na produção, na composição e nas propriedades tecnológicas do leite em diferentes raças leiteiras. Este estudo aplicou a técnica de reação em cadeia da polimerase seguida de análise de polimorfismo de comprimento de fragmentos de restrição (PCR-RFLP) para caracterizar variantes dos genes β-Lg e κ-Cn em bovinos leiteiros das raças Jersey (JE), mestiços (XX) e Crioulos (CR) no Panamá. A variação genotípica e alélica foi avaliada utilizando uma amostra de conveniência composta por 351 amostras de sangue coletadas entre 2014 e 2015 (JE, n = 230; XX, n = 90; CR, n = 26). Embora a estratégia de amostragem não permita inferências em nível populacional, ela é adequada para avaliar a viabilidade da técnica PCR-RFLP e realizar uma caracterização exploratória da variabilidade genética. O sucesso de amplificação foi de 98,6% para β-Lg e de 76,6% para κ-Cn. No locus β-Lg, o alelo A predominou nos animais JE (0,55) e CR (0,52), enquanto o alelo B foi mais frequente nos animais XX (0,73). No locus κ-Cn, o alelo B predominou em todos os grupos avaliados. De modo geral, a técnica PCR-RFLP mostrou-se um método prático e acessível para a detecção de variações em genes de proteínas do leite, apoiando sua aplicação em futuros estudos populacionais conduzidos sob as condições de produção do Panamá.

## Introduction

Sustained profitability in dairy production requires continuous improvements in both milk yield and milk composition. These traits are strongly influenced by genetic factors, which also help determine the most appropriate end use of milk within dairy production systems. In Panama, annual consumption is estimated at approximately 480 million liters of milk, including fluid milk and a variety of domestic and imported dairy products (Instituto Nacional de Estadística y Censo, 2024; Panamá, 2021). However, local production supplies only about 180 million liters, while per capita consumption averages 120 liters per year (Panamá, 2021). This persistent supply deficit underscores the need to strengthen national dairy production to meet growing consumer demand. Panama is home to a diverse range of native and introduced cattle breeds selected for their potential to enhance dairy productivity (Villalobos-Cortés et al., 2012). These breeds possess valuable genetic traits associated with milk production, disease and ectoparasite resistance, and adaptation to tropical environments. Maximizing these advantages requires the implementation of strategies and technologies that support the monitoring and improvement of milk yield while maintaining high-quality production standards. Given the use of a convenience sample, this study is intended as an exploratory characterization rather than a population‑level assessment.

Globally, efforts to improve dairy productivity have included the characterization and selection of polymorphisms in milk protein genes, particularly β-lactoglobulin (β-Lg) and κ-casein (κ-Cn) (Ozdemir et al., 2018). Milk proteins consist of an acid‑precipitable fraction (caseins) and an acid‑soluble fraction (whey proteins), the latter of which includes β-Lg (Kontopidis et al., 2004; Holt et al., 2013). These proteins are encoded by autosomal genes that follow a Mendelian pattern of inheritance, enabling the selection of favorable genetic variants in breeding programs (Aschaffenburg & Drewry, 1955; Ng-Kwai-Hang et al., 1984). Genetic variants of β-Lg, α-lactalbumin, and the four major caseins (αs1, αs2, β, and κ), have been associated with differences in milk yield, composition, and technological properties (Jakob & Puhan, 1992; Fox & McSweeney, 2003). For example, the β‑Lg AA genotype has been associated with higher total protein content, whereas the BB genotype has been linked to increased fat and casein concentrations (Cendron et al., 2021). Similarly, κ-Cn variants strongly influence milk coagulation properties relevant to cheesemaking, including rennet coagulation time, curd firmness, and cheese yield (Ng-Kwai-Hang et al., 1989). In particular, the κ‑Cn BB genotype has been associated with improved rennet coagulation properties, while the AA genotype has been linked to higher protein content (Ng-Kwai-Hang et al., 1984; Lopez, 2004; Cendron et al., 2021).

Genetic characterization of β‑Lg and κ‑Cn has commonly been performed using polymerase chain reaction‑restriction fragment length polymorphism (PCR-RFLP) (Medrano & Aguilar-Cordova, 1990; Meilhoc, 1990). This method enables the detection of allele‑specific sequence variation through PCR amplification followed by restriction enzyme digestion (Saiki et al., 1985). Because PCR‑RFLP is relatively low‑cost, technically simple, and compatible with basic molecular biology infrastructure, it represents a practical approach for exploratory genotyping in agricultural and veterinary research settings with limited resources (Tarach, 2021).

In this context, the present study aimed to evaluate the feasibility of applying PCR‑RFLP to characterize β‑Lg and κ‑Cn polymorphisms and to describe genotype and allele distributions among available dairy cattle DNA samples from Panama. By generating baseline genetic information through an accessible molecular approach, this study contributes to ongoing efforts to assess milk protein polymorphisms that may inform future breeding strategies and support improvements in dairy production under tropical conditions.

## Materials and methods

### Sampling

A total of 351 bovine blood samples were collected between 2014 and 2015 as part of the disease control and surveillance program of the Ministerio de Desarrollo Agropecuario. Sampling included Jersey dairy cattle (JE) from Coclé Province, crossbred dairy cattle (XX), and Creole cattle (CR), following approved National Veterinary Accreditation Program (NVAP)/Animal and Plant Health Inspection Service (APHIS) abbreviation codes (National Veterinary Accreditation Program, 2010). The XX group represented a dual‑purpose production system and consisted primarily of *Bos taurus* × *Bos indicus* crosses, including Zebu × Brown Swiss and Zebu × Holstein crosses. All JE samples originated exclusively from Coclé Province, whereas XX and CR samples were obtained from multiple provinces, including Chiriquí, Veraguas, Herrera, Los Santos, Coclé, and the Ngöbe‑Buglé Comarca. Sampling was conducted using a non‑randomized, convenience‑based approach.

Genotypes at the β-Lg and κ-Cn loci were characterized using a convenience DNA sample set, selected based on availability. Although this sampling approach does not support population-level inference, it is appropriate for evaluating the PCR-RFLP genotyping method and conducting exploratory genetic characterization.

### DNA extraction and PCR-RFLP

Genomic DNA was extracted using ion-exchange purification columns (Qiagen®) and stored at −20 °C until genotyping. Genotyping of the β-Lg and κ-Cn loci was performed by PCR-RFLP, following a modified version of the method described by Medrano and Aguilar-Cordova (1990). For XX and CR cattle, DNA samples were processed for κ‑Cn genotyping approximately 1 year after β‑Lg characterization. In contrast, DNA from JE cattle was analyzed for both loci within approximately 6 months of sample collection. PCR conditions were optimized empirically prior to genotyping. PCR amplification was performed using genomic DNA (<100 ng/μL), a commercial master mix (Promega, Cat. No. M7502), and locus-specific primer pairs.

For the β-Lg locus, primers BGLP3 (5′-GTCCTTGTGCTGGACACCACCGACTACA-3′) and BGLP4 (5′-CAGGACACCCTCTCCGGTATATGA-3′) were used to amplify an approximately 260-bp fragment. For the κ-Cn locus, primers JK5 (5′-ATCATTTATGGCCATTCCAAAG-3′) and JK3 (5′-GCCCATTTCGCCTTCTCTGTAACAGA-3′) were used to amplify an approximately 350-bp fragment. PCR amplification was performed using a thermal cycler (Thermal Cycler 2720, Applied Biosystem®). For the β-Lg locus, cycling conditions consisted of an initial denaturation at 94 °C for 3 min; 35 cycles of denaturation at 94 °C for 45 s, annealing at 60 °C for 45 s, and extension at 72 °C for 1 min; followed by a final extension at 72 °C for 10 min. For the κ-Cn locus, conditions included an initial denaturation at 94 °C for 3 min; 45 cycles of denaturation at 94 °C for 30 s, annealing at 57 °C for 30 s, and extension at 72 °C for 30 s; followed by a final extension at 72 °C for 10 min. PCR products were resolved on 1.5% agarose gels stained with ethidium bromide and visualized under ultraviolet illumination using a photo-documentation system (Ultra-Lum®).

**Figure 1:**
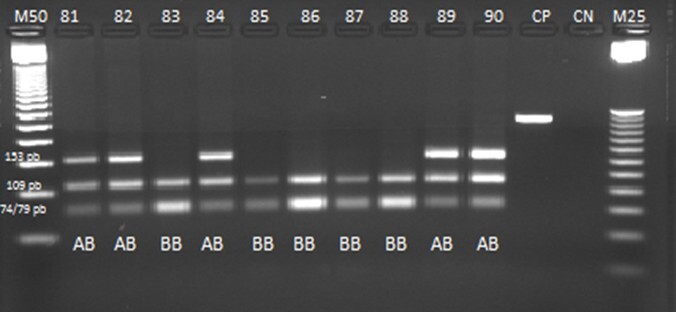
**PCR–RFLP genotyping of the bovine β‑lactoglobulin β-Lg gene**. Lanes 81–90 show digestion patterns corresponding to genotypes AB and BB. Digestion with *HaeIII* yielded fragments of 153, 109, and 74/79 bp for the AB genotype, and yielded only the 109 and 74/79 bp fragments for the BB genotype. M50 and M25 denote molecular size markers. CP and CN represent positive and negative controls, respectively.

**Figure 2:**
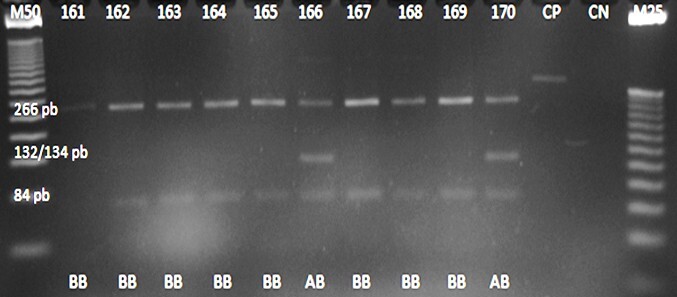
**PCR–RFLP genotyping of the bovine κ- Casein (κ-Cn) gene**. Lanes 161–170 show digestion patterns corresponding to genotypes AB and BB. Digestion with *HinfI* yielded fragments of 266, 132/134, and 84 bp for the AB genotype, and yielded only the 266 and 84 bp fragments for the BB genotype. M50 and M25 denote molecular size markers. CP and CN represent positive and negative controls, respectively.

Amplified products were subjected to RFLP analysis using *HaeIII* for β‑Lg and *HinfI* for κ‑Cn (Promega®), following the manufacturer’s recommendations. Digested fragments were separated on 3% agarose gels, and genotype‑specific banding patterns were interpreted according to previously published criteria. Figures 1 and 2 present the representative electrophoretic profiles for each genotype.

### Statistical analysis

All statistical analyses were conducted in R (version 4.5.0; R Core Team, 2025). Associations between breed and genotype at the β‑Lg and κ‑Cn loci were evaluated using contingency‑table analyses and Pearson’s chi‑square (χ^2^) test, with all expected cell counts meeting the assumption of ≥ 5. Effect sizes were quantified using Cramér’s V, and standardized χ^2^ residuals were examined to identify genotype categories contributing most strongly to significant associations. When significant overall heterogeneity was detected, post‑hoc pairwise comparisons among breeds were performed using Fisher’s exact tests with Holm-adjusted p-values to account for multiple comparisons. Hardy–Weinberg equilibrium (HWE) was assessed separately within each breed for both loci.

## Results

DNA samples from 351 cows were analyzed for polymorphisms in the β-Lg and κ-Cn milk protein genes. Of these, 267 samples (76%) were successfully amplified by PCR for both loci. The β-Lg locus was amplified in 346 of 351 samples (98.6%), whereas the κ-Cn locus was amplified in 269 of 351 samples (76.6%) (Table 1). For β-Lg, PCR amplification was successful in 230 of 232 JE samples (99.1%), 90 of 92 XX samples (97.8%), and 26 of 27 CR samples (96.3%), yielding an amplicon of approximately 260 bp (Table 1). Similarly, the κ-Cn locus was successfully amplified in 210 of 232 JE samples (90.5%), 42 of 92 XX samples (45.7%), and 17 of 27 CR samples (63%), generating an amplicon of approximately 350 bp (Table 1).

**Table 1:** Description of cow groups and the number of genotype-positive animals in each locus.

**Breed of dairy cow**	**No of Animals (%)**	**Genotype‑positive animals, n (%)**	
		β**-LG**	**k-CN**
Jersey (JE)	232 (66.1)	230 (99.1)	210 (90.5)
Crossbred (XX)	92 (26.2)	90 (97.8)	42 (45.7)
Creole (CR)	27 (7.7)	26 (96.3)	17 (63.0)
			
Total	351 (100)	346 (98.6)	269 (76.6)

Genotype frequencies at the β-Lg locus differed significantly among breeds (χ^2^ = 38.9, df = 4, *P*‑value = 7.3 × 10^−8^; Cramér’s V = 0.237), indicating a small effect size. The AB genotype predominated in JE and CR cattle (0.47 and 0.58, respectively), whereas XX cattle showed a higher frequency of the BB genotype (0.54) (Table 2; Supplementary Table S1). Post‑hoc pairwise comparisons indicated no significant difference between JE and CR cattle, whereas both differed significantly from XX cattle after Holm adjustment. No deviations from HWE were detected at the β‑Lg locus in any breed.

**Table 2:** Distribution of polymorphisms of genotypes and alleles of the β-Lg and κ-Cn milk protein genes.

**Gene**	**Genotype/Allele**	**Frequency of genotypes and alleles**			
		**JE (n=230)**	**XX (n=90)**	**CR (n=26)**	***P-*value** a
β-Lg	AA	0.31	0.09	0.23	7.3 × 10^-8^
	AB	0.47	0.37	0.58	
	BB	0.22	0.54	0.19	
	A	0.55	0.27	0.52	2.9 × 10^−9^
	B	0.45	0.73	0.48	
κ-Cn	AA	0.05	0.24	0.12	8.7 × 10^-7^
	AB	0.20	0.36	0.53	
	BB	0.75	0.40	0.35	
	A	0.15	0.42	0.38	8.1 × 10^−9^
	B	0.85	0.58	0.62	

a *P-value* obtained from a chi‑square (χ^2^) test comparing genotype frequencies and allele frequencies among JE, XX, and CR groups.

Allele frequencies at the β‑Lg locus also differed significantly among breeds (χ^2^ = 39.3, df = 2, *P*‑value = 2.9 × 10^−9^; Cramér’s V = 0.238). Allele A was more frequent than allele B in JE and CR cattle (0.55 and 0.52, respectively), whereas allele B predominated in XX cattle (0.73).

Genotype distributions at the κ‑Cn locus varied significantly across breeds (χ^2^ = 33.6, df = 4, *P*‑value = 8.7 × 10^−7^; Cramér’s V = 0.250). The BB genotype was most frequent in JE (0.75) and XX (0.40) cattle, whereas CR cattle showed a higher frequency of the AB genotype (0.53) (Table 2; Supplementary Table S2). Pairwise comparisons indicated that XX cattle differed significantly from both JE and CR cattle, whereas JE and CR did not differ significantly from each other. A significant deviation from HWE was observed in JE cattle at the κ‑Cn locus (*P*‑value = 0.0043), characterized by a heterozygote deficit, while the XX and CR populations conformed to HWE expectations.

Allele frequencies at the κ‑Cn locus differed significantly among breeds (χ^2^ = 37.3, df = 2, *P*‑value = 8.1 × 10^−9^; Cramér’s V = 0.238). Allele B predominated across all groups, with the highest frequency in JE cattle (0.85), followed by CR (0.62) and XX cattle (0.40).

## Discussion

In this study, PCR-RFLP was used to differentiate among the three genotypic classes (AA, BB, and AB) of the β-Lg and κ-Cn genes, as originally described by Medrano and Aguilar-Cordova (1990). The approach enabled the identification of allele frequencies and genotype distributions within a convenience sample. Overall, the results demonstrate that PCR-RFLP is a reliable and practical method for assessing the genetic background of dairy cattle.

Regarding genotyping results, the β‑Lg allele A predominated in JE and CR cattle, consistent with the higher frequency of the AB genotype, followed by the AA genotype in these groups. In heterozygous animals, allele A is typically expressed more strongly than allele B, with AB cows producing a higher proportion of β‑Lg A relative to β-Lg B. For instance, in a large breed‑level comparative study, allele A exceeded allele B expression by 49% in JE cattle and 41% in Canadienne cattle, demonstrating that the functional dominance of allele A is not only genotypic but also reflects higher protein output at the phenotypic level (Ng-Kwai-Hang & Kim, 1996). In contrast, XX cattle in our study showed a clear predominance of allele B, reflecting the overrepresentation of BB genotypes identified through standardized residual analysis. These genotypic patterns align with previously reported functional differences between β‑Lg alleles, where allele A has been associated with slightly higher whey protein content, whereas allele B has been linked to improved casein yield and favorable cheesemaking properties (Lunden et al., 1997; Ikonen et al., 1999; Cendron et al., 2021).

For the κ-Cn gene, a high frequency of allele B was observed across JE, XX, and CR cattle, consistent with the predominance of BB genotypes in JE and XX cattle, and AB in CR cattle. In previous studies, the κ‑Cn B allele has been associated with higher casein content and improved cheesemaking properties, including greater curd firmness and shorter rennet coagulation time (Cendron et al., 2021). A higher frequency of this allele has also been reported in Limonero CR cattle from Venezuela and tropical dairy CR populations from Mexico (Rojas et al., 2009; Becerril-Pérez et al., 2020). In contrast, studies of CR cattle in Peru and Argentina have reported a predominance of allele A, corresponding to a higher frequency of AA genotypes in those populations (Poli et al., 2005; Veli & Rivas, 2010). Although these results are not intended as population-level estimates, the observed allele distributions indicate that the PCR‑RFLP protocol reliably captures expected genetic variation among cattle groups, even under basic laboratory conditions.

The PCR-RFLP analysis also revealed a high frequency of heterozygous (AB) genotypes for both β-Lg and κ-Cn genes in CR cattle. From an exploratory perspective, such genetic profiles may offer flexibility for future breeding strategies aimed at balancing alleles associated with milk production traits (e.g., β‑Lg A) and cheesemaking properties (e.g., κ‑Cn B). In CR cattle, the observed heterozygosity may also reflect broader genetic diversity, which in other studies has been associated with improved adaptability to local environmental stressors, including climate resilience, disease resistance, and performance in diverse ecological systems (Villalobos-Cortés et al., 2012; Dikmen et al., 2015; Tempel Stumpf et al., 2021; Campos et al., 2022; Ben-Jemaa et al., 2024). However, these adaptive traits were not directly assessed in the present study; therefore, any inferred associations should be considered hypothetical.

As noted in the Methods section, our study relied on convenience sampling rather than a population‑based design; the allele frequencies observed for β‑Lg and κ‑Cn should be interpreted as illustrative outcomes of the PCR‑RFLP workflow rather than estimates of breed‑level genetic structure. Despite these limitations, the frequencies observed across the three cattle groups were biologically coherent and aligned with the genotype distributions obtained through PCR‑RFLP, supporting the reliability of the method.

The lower amplification success observed for κ‑Cn relative to β‑Lg may be attributed to locus‑specific technical challenges. PCR‑RFLP assays targeting the κ‑Cn gene typically involve guanine–cytosine‑rich and structurally complex genomic regions, which are more sensitive to DNA quality and amplification conditions (Medrano & Aguilar-Cordova, 1990). In addition, differences in sample processing timelines and storage conditions among breed groups may have exacerbated these effects, particularly for technically demanding targets such as κ‑Cn (Al-Soud & Radstrom, 2001).

Overall, this study strengthens the methodological capacity for the genetic characterization of locally available cattle populations and provides preliminary allele‑frequency data that may support future population‑based studies, breeding strategies, and the conservation of locally adapted genetic resources under Panamanian production conditions.

## Conclusions

Overall, PCR‑RFLP provides a practical and accessible approach for identifying β‑Lg and κ‑Cn genotypes in dairy cattle under resource‑limited laboratory conditions in Panama. The allele‑frequency patterns described here should be interpreted as exploratory baseline data derived from convenience sampling. Nevertheless, these findings support future efforts aimed at developing more accessible or field‑adapted genotyping strategies and at gradually integrating genetic markers into breeding and selection programs. A coordinated, nationwide evaluation involving academic and regulatory collaborators would further strengthen genetic improvement initiatives and help align dairy production systems with economically optimal uses of milk.

## Supplementary Material

Supplementary material accompanies this paper.

Supplementary table 1: Genotype and allele frequencies (with 95% confidence intervals) by breed for β‑lactoglobulin (β-Lg).

Supplementary table 2: Genotype and allele frequencies (with 95% confidence intervals) by breed for κ‑casein (κ-Cn).

This material is available as part of the online article from https://doi.org/10.29374/2527-2179.bjvm001026
