# Supplementary material for: Characterization of β‑lactoglobulin and κ‑casein genotypes PCR-RFLP in dairy cattle from Panama
Source: Braz J Vet Med. 2026 Jul 24;48:e001026. doi: 10.29374/2527-2179.bjvm001026 (PMC13399929; doi:10.29374/2527-2179.bjvm001026)
Supplement: Supplementary table 1: [file bjvm-48-e001026-suppl.pdf]

Supplementary table 1: Genotype and allele frequencies (with 95% confidence intervals) by breed for β-lactoglobulin (β-Lg).

Breed	Metric	Count	N	Freq	CI_exact_low	CI_exact_high	CI_wilson_low	CI_wilson_high
JE	β-Lg Genotype: AA	72	230	0.31	0.25	0.38	0.25	0.38
JE	β-Lg Genotype: AB	107	230	0.47	0.4	0.53	0.4	0.53
JE	β-Lg Genotype: BB	51	230	0.22	0.17	0.28	0.17	0.28
JE	β-Lg Allele: A	251	460	0.55	0.5	0.59	0.5	0.59
JE	β-Lg Allele: B	209	460	0.45	0.41	0.5	0.41	0.5
XX	β-Lg Genotype: AA	8	90	0.09	0.04	0.17	0.04	0.17
XX	β-Lg Genotype: AB	33	90	0.37	0.27	0.47	0.27	0.48
XX	β-Lg Genotype: BB	49	90	0.54	0.44	0.65	0.44	0.65
XX	β-Lg Allele: A	49	180	0.27	0.21	0.34	0.21	0.34
XX	β-Lg Allele: B	131	180	0.73	0.66	0.79	0.66	0.79
CR	β-Lg Genotype: AA	6	26	0.23	0.09	0.44	0.1	0.44
CR	β-Lg Genotype: AB	15	26	0.58	0.37	0.77	0.37	0.76
CR	β-Lg Genotype: BB	5	26	0.19	0.07	0.39	0.07	0.4
CR	β-Lg Allele: A	27	52	0.52	0.38	0.66	0.38	0.66
CR	β-Lg Allele: B	25	52	0.48	0.34	0.62	0.34	0.62

Supplementary table 2: Genotype and allele frequencies (with 95% confidence intervals) by breed for κ-casein (κ-Cn).

Breed	Metric	Count	N	Freq	CI_exact_low	CI_exact_high	CI_wilson_low	CI_wilson_high
JE	κ-Cn Genotype: AA	10	210	0.05	0.02	0.09	0.02	0.09
JE	κ-Cn Genotype: AB	43	210	0.2	0.15	0.27	0.15	0.27
JE	κ-Cn Genotype: BB	157	210	0.75	0.68	0.8	0.68	0.8
JE	κ-Cn Allele: A	63	420	0.15	0.12	0.19	0.12	0.19
JE	κ-Cn Allele: B	357	420	0.85	0.81	0.88	0.81	0.88
XX	κ-Cn Genotype: AA	10	42	0.24	0.12	0.39	0.13	0.4
XX	κ-Cn Genotype: AB	15	42	0.36	0.22	0.52	0.22	0.52
XX	κ-Cn Genotype: BB	17	42	0.4	0.26	0.57	0.26	0.57
XX	κ-Cn Allele: A	35	84	0.42	0.31	0.53	0.31	0.53
XX	κ-Cn Allele: B	49	84	0.58	0.47	0.69	0.47	0.69
CR	κ-Cn Genotype: AA	2	17	0.12	0.01	0.36	0.02	0.38
CR	κ-Cn Genotype: AB	9	17	0.53	0.28	0.77	0.29	0.76
CR	κ-Cn Genotype: BB	6	17	0.35	0.14	0.62	0.15	0.61
CR	κ-Cn Allele: A	13	34	0.38	0.22	0.56	0.23	0.56
CR	κ-Cn Allele: B	21	34	0.62	0.44	0.78	0.44	0.77
